# Coupling fullerene into porous aromatic frameworks for gas selective sorption†
†Electronic supplementary information (ESI) available: Experimental details and characterizations. See DOI: 10.1039/c6sc00134c


**DOI:** 10.1039/c6sc00134c

**Published:** 2016-02-22

**Authors:** Ye Yuan, Peng Cui, Yuyang Tian, Xiaoqin Zou, Yingxi Zhou, Fuxing Sun, Guangshan Zhu

**Affiliations:** a Key Laboratory of Polyoxometalate Science of the Ministry of Education , Faculty of Chemistry , Northeast Normal University , Changchun , 130024 , P. R. China . Email: zhugs100@nenu.edu.cn; b State Key Laboratory of Inorganic Synthesis and Preparative Chemistry , Jilin University , Changchun , 130012 , P. R. China

## Abstract

A lot of investigations calculate that fullerene would be an excellent unit for porous adsorbents for gas sorption and separation. Although the conjugated structure and symmetrical shape of fullerene makes it an ideal building block, there is no facile strategy to achieve fullerene-based porous materials. Herein, we adopt a novel acid catalyzed coupling reaction to bind the fullerene molecules together in a one-step synthesis. After synthesizing the adducts as models, the atomic connectivity between the fullerene molecules is determined clearly. We then prepared a series of fullerene-based porous aromatic frameworks (PAFs). The targeted products adsorb H_2_ and CO_2_, and they exhibit some selectivity of CO_2_ over N_2_ and CH_4_. The good performance of the PAFs indicates that we could combine the gas binding ability of the building block and the porous nature of PAF materials together to give a better adsorbent.

## Abstract 2

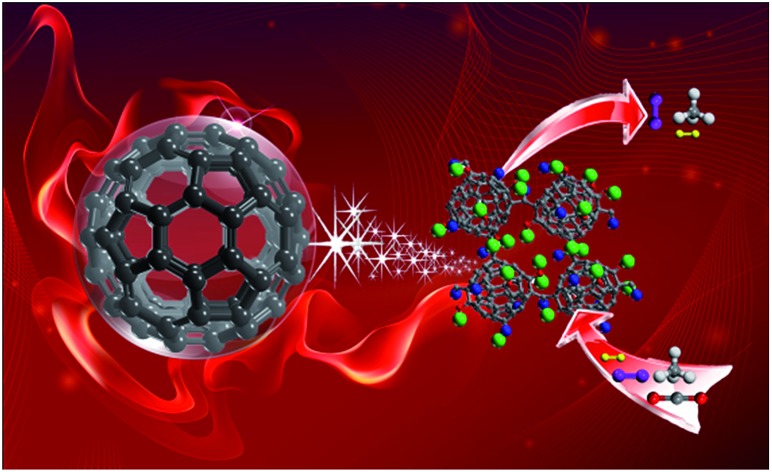
Fullerene molecules were connected to form fullerene-based PAFs. The porous structure could adsorb H_2_ and exhibit some selectivity of CO_2_.

## Introduction

Fullerene and its derivatives have attracted worldwide attention. Their high conjugate structures are favourable for ultra-fast electron transfer and therefore they have been intensively studied in solar cells,^1^ superconductors,^2^ and ferromagnetic materials.^3^ Recently, many theoretical studies predicted that fullerene and its derivatives may provide a popular environment for gas sorption or storage, including calculation combined DFT and *ab initio* molecular dynamics predicting that the maximum hydrogen capacity of C60 is approximately 7.5 wt% (H58@C60), and that there are three different adsorption sites in C60 molecules for CO_2_.^4^ If fullerene compounds are used as novel porous adsorbents, they may reveal superior performance in gas sorption and separation for their excellent gas binding interactions.^5^ However, the sad fact is that pure fullerene is tightly packed and possesses no pores or cavities. Thus, a lot of work is focused on building fullerene-based extended networks or intercalating fullerene compounds into porous frameworks. The earliest investigation was on a fullerene-containing dimer synthesized by the connection of pyridyl groups and Pt^II^ ions.^6^ Since then, many other strategies have been investigated, such as, designing fullerene derivatives for coordination structures^7^ or trapping fullerene into pores and cavities.^8^ Though the conjugated structure and symmetrical shape of fullerene make it an ideal building unit for constructing a porous skeleton, no facile method to achieve fullerene-based porous materials has been reported.

Porous organic frameworks (POFs), being famous for their all organic components, have attracted significant interest in catalysis, gas storage, energy conversion, and optoelectronics.^9^ The first POF material, named polymers of intrinsic microporosity (PIM), was reported by Budda *et al.*^10^ Their developments have been greatly accelerated by the emergence of covalent organic frameworks (COFs)^11^ and porous organic cages (CC).^12^ Porous aromatic frameworks (PAFs), as a subclass of porous organic frameworks, have made another progress in the design and synthesis of unique porous skeletons.^13^ The high stability and proper pore sizes provide even more possibilities for energy gas sorption and separation. Besides, their multiple methodologies for organic coupling reactions ensure various construction strategies to prepare diverse structures.

In this article, *via* a one-step acid catalyzed coupling reaction, C60 was adopted as a novel rigid spherical building block to construct PAFs. We first prepared the fullerene-based adducts as a model to verify the atomic connection method during the coupling reaction. Subsequently, we investigated the structural variations of the PAFs obtained with various dimethoxy compounds at different raw material ratios. Detailed characterizations of high resolution transmission electron microscopy (HR-TEM), pore size distribution and energy minimization optimization suggest a possible chemical fragment of fullerene-based PAFs. Besides their chemical and thermal stabilities, fullerene-based PAFs possess high surface areas. As expected, the fullerene-based PAFs combine the gas binding ability of C60 and the porous nature of PAFs. They exhibit a good adsorption capacity for H_2_ and CO_2_, and have a relatively high CO_2_ selectivity.

## Results and discussion

**Fig. 1:**
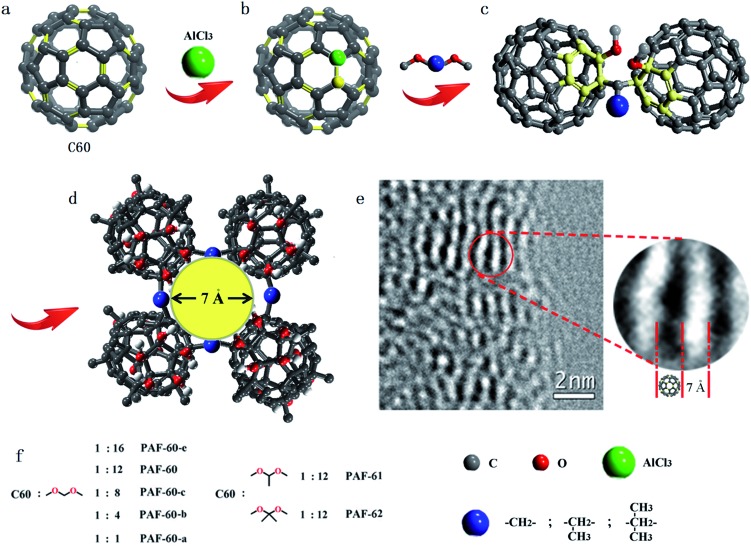
(a) Fullerene, (b) and (c) the main coupling methods, (d) the possible fragments based on pore-size distribution and energy minimization optimization calculated by Materials Studio in order to guide the eyes, (e) high resolution TEM image of PAF-60, and (f) PAF-60-a, PAF-60-b, PAF-60-c, PAF-60, and PAF-60-e synthesized by C60 and dimethoxy methane in different molar ratios, PAF-61 and PAF-62 synthesized by C60 and other dimethoxy compounds (blue balls represent different alkyl groups).

Fig. 1 illustrates a scheme of the preparation process for fullerene-based PAF materials. Typically, PAF-60-a, PAF-60-b, PAF-60-c, PAF-60, and PAF-60-e were synthesized by the reaction of dimethoxy methane and C60 in different molar ratios (1 : 1, 4 : 1, 8 : 1, 12 : 1, and 16 : 1, respectively) at 150 °C in nitrobenzene for 72 h. The optimized ratio (dimethoxy methane to C60 of 12 : 1) was confirmed by assessing the surface areas and gas sorption ability of the PAFs. Acetaldehyde dimethyl acetal and 2,2-dimethoxypropane were also carefully selected as linkers to afford PAF-61 and PAF-62, respectively.

As reported, the double bond of the fullerene molecule could be broken under the presence of an AlCl_3_ catalyst and the neutral fullerene molecule evolved to a fullerene cation, followed by electrophilic fullerenation of the aromatics (Fig. S1a†).^14^ Then, the fullerene cation could react with various dimethoxy compounds. To probe the possible reaction mechanism and atomic connectivity, we chose dimethoxy methane and fullerene as reactants to prepare the fullerene-based methoxy monoadduct (Fig. S1†) and dimethoxy connected dimer (Fig. S2†). After a great deal of experiments and iteratively purifying, we obtained the two pure fullerene compounds. As shown in Fig. S1,† the ^1^H nuclear magnetic resonance (NMR) spectrum of the monoadduct in solution reveals three main signals [*δ* 4.24 (d, *J* = 6.8 Hz, 2H), 3.76 (s, 3H), and 3.21 (s, 3H)], which correspond to the three kinds of H atoms (–CH_2_–, C60–O–CH_3_ and –O–CH_3_, respectively) in the methoxyl monoadduct. Also, the ^13^C NMR spectrum shows a group of signals (*δ* 50.58, 59.79, 67.48 and ∼143) associated with the three alkyl carbons of dimethoxy compounds and the conjugated carbons of C60, respectively. The C–H bands (–CH_2_–) at 2863 and 2946 cm^–1^, (–CH_3_) at 2846 and 2916 cm^–1^, and C–O band at 1099 cm^–1^ in the Fourier-transform infrared (FTIR) spectrum demonstrates the chemical bonding of the methoxyl monoadduct and the peak of the molecular weight in the HPLC-ESI/MS spectrum, centered at 796, could verify its generation. As for the dimethoxy connected dimer, it is poorly soluble in common solvents, but the peak of the molecular weight located at 1516 could help to indicate its existence (Fig. S2†). In the meantime, due to the existence of H_2_O and AlCl_3_, the methoxyl group in the methoxyl monoadduct may generate into a hydroxyl group and the methoxyl monoadduct would turn into a hydroxyl monoadduct (Fig. S3†), which is proven by IR and MS characterizations.^15^ All of the above evidence could help us to confirm their coupling methods.

A series of PAFs (PAF-60-a, PAF-60-b, PAF-60-c, PAF-60, and PAF-60-e) with different molar ratios of pristine reactants, were prepared to verify the changes in the structure during the coupling process. PAF-61 and PAF-62 were synthesized as well in order to tune the porous nature of PAF-60 by increasing the volume of side methyl substituents. FTIR (Fig. S4†), solid-state ^13^C NMR (Fig. S5†) and elemental analysis (Table S1†) were used to identify their bonding models. With similar band positions for the fullerene-based methoxy adducts, the C–H vibrations [(–CH_2_–) at around 2861 and 2943 cm^–1^, (–CH_3_) at 2845 and 2916 cm^–1^] and C–O band (1099 cm^–1^) of the polymeric frameworks become increasingly obvious with more dimethoxy methane being added to the reaction system. Simultaneously, the relative intensity of C=C (1425 cm^–1^) is reduced gradually, proving the degradation of the conjugation of the original C60.^16^ For PAF-61 and PAF-62, the C–H vibrations [(–CH_3_) at 2845 and 2916 cm^–1^] become much stronger than those of PAF-60, in good agreement with their high methyl content in pristine reactant. Solid-state ^13^C NMR (Fig. S5†) studies reveal two main signals at 0–70 and 100–170 ppm, which are assigned to the alkyl carbons of dimethoxy compounds and the conjugated carbons of C60, respectively. As shown in Table S1,† the elemental analysis confirmed their C and H content, which was used to estimate the ratio of reactants in the infinite PAF sheet. For PAF-60-a, PAF-60-b, PAF-60-c, PAF-60, and PAF-60-e, the H content increases as the dimethoxy methane scaled up and the rate finally levelled off. These results further verify that the coupling reaction in Fig. 1 occurs as expected. Judging from the C/H ratios determined by the elemental analysis (Table S1†), it can be concluded that PAF-61 and PAF-62 have a similar stoichiometry of dimethoxy precursor to C60 (12 : 1). This finding, together with the IR data, proves that PAF-61 and PAF-62 possess the same chemical structure as PAF-60.

The powder X-ray diffraction (PXRD) patterns (Fig. S6†) indicate that all of the fullerene-based PAFs are amorphous. No long-range ordered structure of PAFs could be obtained due to the distortion and interpenetration of flexible dimethoxy linkers. Scanning electron microscopy (SEM) images reveal that the PAFs are composed of agglomerates of sub-micrometer size (Fig. S7†). Transmission electron microscopy (TEM) images show that the PAFs have amorphous porous textures in the long range order (Fig. S8†). However, local order structures are preserved, which can be seen from the obvious fingerprint texture, with a value of around 0.7 nm, in the HR-TEM image as shown in Fig. 1e. This value of 0.7 nm matches well with the molecular size of C60 (0.71 nm), which sheds light on the intact C60 structure in PAF-60. Thermogravimetric analysis (TGA) was performed in a dry air atmosphere to probe the thermal stability and purity of the PAF materials. The result displayed in Fig. S9† suggests that the decomposition of the skeletons of all PAFs takes place over 200 °C, and there remains hardly any residue at 600 °C for each PAF material. The negligible weight loss after immersing the PAF samples in a variety of polar and non-polar organic solvents (*e.g.* methanol, acetone, chloroform, toluene and hexane) indicates that the prepared PAFs have good chemical stabilities. ICP analysis was performed for possible residues of Al^3+^ cations. The low content of 1.1–3.3% in all PAFs clearly shows that the PAF-60, PAF-61 and PAF-62 materials are in a pure phase, in accordance with the TGA analysis (Table S1†).

**Fig. 2:**
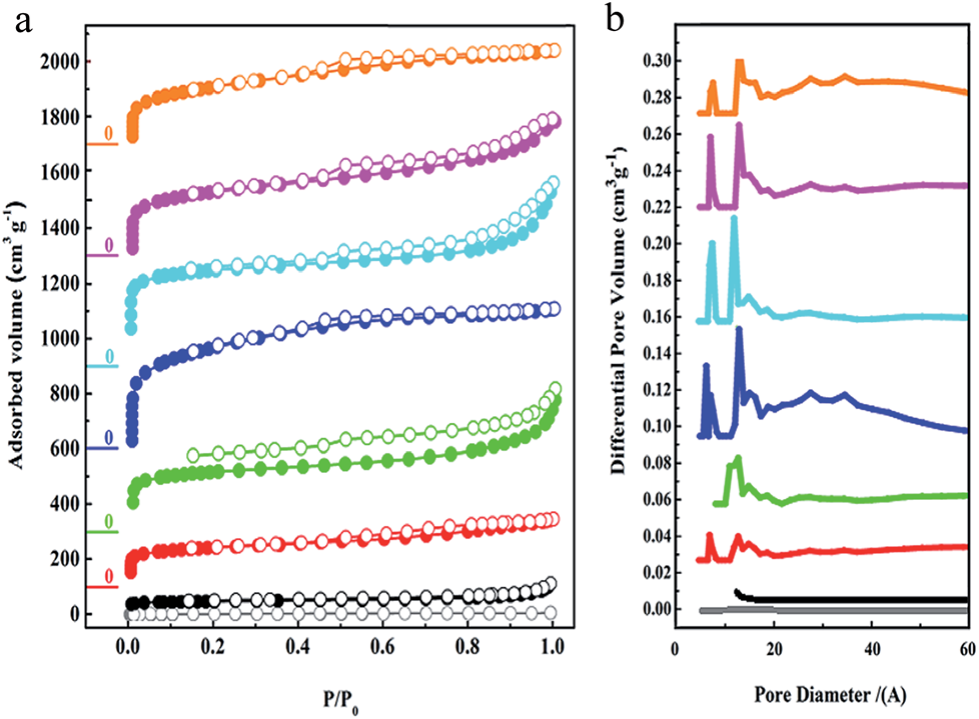
(a) Nitrogen gas adsorption isotherms measured at 77 K and STP, and (b) the curves of the pore size distributions calculated *via* the NL-DFT method for C60 (gray), PAF-60-a (black), PAF-60-b (red), PAF-60-c (green), PAF-60 (blue), PAF-60-e (cyan), PAF-61 (purple), and PAF-62 (orange) from the bottom to the top.

**Fig. 3:**
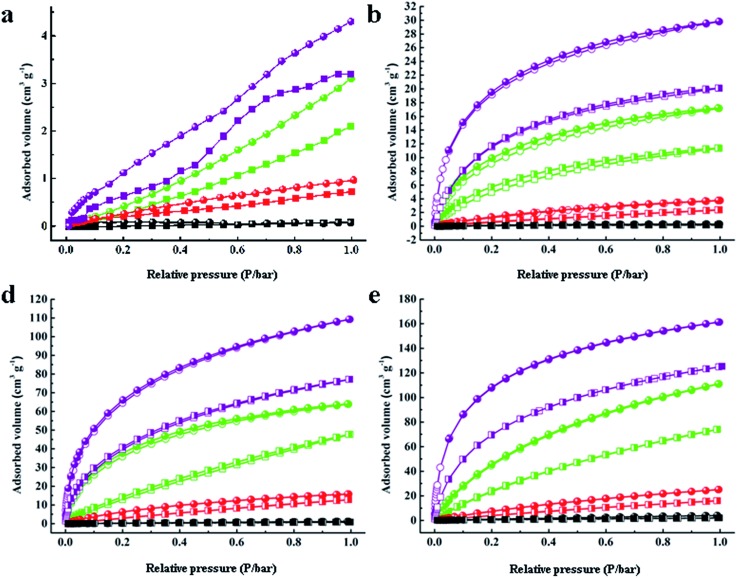
Gas adsorption–desorption isotherms of H_2_, N_2_, CH_4_ and CO_2_ for (a) C60, (b) PAF-60, (c) PAF-61, and (d) PAF-62.

The porous characteristics of all of the PAFs were examined using N_2_ sorption studies at 77 K (Fig. 2). As observed, the apparent Brunauer–Emmet and Teller (BET) surface area of C60 is about 19 m^2^ g^–1^ which demonstrates its tightly packed structure. For the cross-linked fullerenes of PAF-60, they reveal typical type-I adsorption isotherms, indicating their microporous feature. The hysteresis at the high relative pressures (0.6 < *P*/*P*_0_ < 1.0) manifests the existence of mesoporosity, which could stem from the voids between the PAF particles *via* aggregation. The BET surface areas of PAF-60-a to PAF-60 increase greatly from 94 to 1094 m^2^ g^–1^ (Table S1†) with the ratios of dimethoxy methane to C60, suggesting that more dimethoxy methane is effectively involved in linking individual C60 molecules into a porous entity. An optimal ratio of 12 : 1 was obtained for producing highly porous PAF-60, evidenced by the largest surface area of 1094 m^2^ g^–1^ for PAF-60. A slight decrease in surface area for PAF-60-e (852 m^2^ g^–1^) is also observed with continuously increasing the ratio of dimethoxy methane to C60 (16 : 1) in the reaction precursor, which sheds light on a small contribution of dimethoxy methane in coupling dimethoxy methane with C60 in PAF-60-e after exceeding the optimal ratio (12 : 1). According to the high surface area of 1094 m^2^ g^–1^ for PAF-60, we believe that we have expanded the close-packed fullerene molecules to the porous structure successfully if we take neat C60 as a reference (19 m^2^ g^–1^). As expected, the surface areas of PAF-61 and PAF-62 (793 and 701 m^2^ g^–1^) are reduced with the increase of the volume of side methyl substituents. The pore size distributions analyzed by the non-localized density functional theory (NLDFT) indicate that they possess similar pores which are predominantly distributed around 0.7 and 1.2 nm (Fig. 3b). It is worth noting that PAF-60, PAF-61 and PAF-62 display almost the same pore size distribution and only their pore volumes are a little bit different, which suggests they may have identical porous structures. The pore size of 0.7 nm is consistent with the interspace measured from the high resolution TEM image (Fig. 1e).

The sorption affinity was further examined by the adsorption measurements toward different gases. As shown in Fig. 3, PAF-60, PAF-61, and PAF-62 revealed similar adsorption isotherms. A small rise could be observed at low relative pressures followed by a tardy growth and eventually levelling off. The sorption amounts of PAF-60, PAF-61, and PAF-62 are much higher than pure C60 molecules because of their enlarged open surface area compared to C60 (Table 1). The isosteric heats (*Q*_st_) were calculated to evaluate the interaction between the PAF skeleton and gas molecules. The *Q*_st_ values for H_2_ (∼7.6 kJ mol^–1^), CH_4_ (∼24.1 kJ mol^–1^), and CO_2_ (∼34.2 kJ mol^–1^) at a low coverage are almost the same as C60 (7.4 kJ mol^–1^, 23.9 kJ mol^–1^, and 32.4 kJ mol^–1^ for H_2_, CO_2_, and CH_4,_ respectively) (Table S1†). No loss in *Q*_st_ values further supports the conclusion that the fullerene-based PAFs have nearly the same gas sorption affinity to C60 after C60 is coupled with dimethoxy methane into an integrated structure.

**Table 1:** Sorption results for C60 and each PAF including surface areas, sorption capacities and sorption selectivity^*a*^

Name	Surface area (m^2^ g^–1^)	Capacity (cm^3^ g^–1^)	Capacity (cm^3^ g^–1^)			Selectivity (Henry's law)		Selectivity (IAST)	
		H_2_	CO_2_	CH_4_	N_2_	CO_2_/CH_4_	CO_2_/N_2_	CO_2_/CH_4_	CO_2_/N_2_
C60	19	**4.3**/*3.2*	**3.1**/*2.1*	**0.97**/*0.72*	**0.08**/*0.06*	**6.97**/*7.91*	**90.8**/*73.3*	**5.66**/*7.19*	**82.4**/*68.9*
PAF-60	1094	**161**/*125*	**111**/*74*	**25**/*15*	**3.9**/*1.9*	**9.81**/*8.48*	**80.4**/*62.6*	**15.3**/*9.72*	**143**/*91.4*
PAF-61	793	**127**/*100*	**80**/*50*	**19.3**/*14.7*	**3.0**/*2.5*	**10.5**/*8.42*	**64.2**/*36.0*	**12.7**/*10.7*	**64.7**/*39.4*
PAF-62	701	**119**/*89*	**74**/*48*	**13**/*10.7*	**1.3**/*0.8*	**18.7**/*14.4*	**275**/*184*	**13.9**/*11.4*	**250**/*147*

^*a*^The surface area and pore size were calculated by N_2_ sorption isotherms, and interpreted by the BET theory and NLDFT method; H_2_ sorption was measured at 77 K (bold) and 87 K (italics) at 1 atm; N_2_, CO_2_, and CH_4_ sorption was measured at 273 K (bold) and 298 K (italics) at 1 atm. The sorption values of C60 are approximate because of its low surface area.

To study their separation potential, Henry's law and the ideal adsorption solution theory (IAST) were applied to validate the interactions between the adsorbent and the adsorbate. The IAST method could predict the gas adsorption amount of each component from a mixture.^17^ The selectivity of each fullerene-based PAF for CO_2_/CH_4_ and CO_2_/N_2_ was calculated for a 50 : 50 (in volume) gas mixture at 273 K and 298 K, respectively. According to the results calculated by Henry's law, the CO_2_/N_2_ and CO_2_/CH_4_ selectivities of PAF-62 reach 275 and 18.7 at 273 K, respectively, which are much higher than those of the pure C60 molecules (CO_2_/N_2_ and CO_2_/CH_4_ selectivities of 90.8 and 6.97), suggesting that CO_2_ is preferentially adsorbed in PAF-62 pores (Table 1 and Fig. S10†). These results present that the novel PAF networks have a strong affinity toward CO_2_ over N_2_ and CH_4_. The most possible explanation for the high selectivity could be that the dipole–quadrupole interactions between the rest of the π conjugated fragments of fullerene and CO_2_ molecules enhance the affinity.13*a* This performance is much better than some porous materials, such as porous organic frameworks and zeolite imidazolate frameworks (listed in Table S2†).^18^

## Conclusions

In summary, for the first time, C60 was adopted as novel spherical building block to construct fullerene-based PAFs *via* a one-step coupling reaction. After preparing various adducts as models, the atomic connectivity between the fullerene molecules was determined clearly. The series of PAFs could possess various pore characteristics by conditioning the molar ratios of the reactants and adjusting their dimethoxy substituents. As designed, the targeted products not only extend the surface area from C60 molecules, but also keep their affinity to gases. The fullerene-based PAFs could absorb a certain amount of H_2_ and CO_2_ molecules, and PAF-60, PAF-61 and PAF-62 exhibit preferred adsorption for CO_2_, thus making them promising in gas separation and purification. Apart from the adsorption properties, the success of constructing PAFs in one step opens a novel strategy for facile preparation of fullerene-based polymers and complexes.

## Supplementary Material

Supplementary information
