# Supplementary material for: Coupling fullerene into porous aromatic frameworks for gas selective sorption
Source: Chem Sci. 2016 Feb 22;7(6):3751–6. doi: 10.1039/c6sc00134c (PMC6008602; doi:10.1039/c6sc00134c)
Supplement: Supplementary file 1 [file SC-007-C6SC00134C-s001.pdf]

Electronic Supplementary Material (ESI) for Chemical Science.
This journal is © The Royal Society of Chemistry 2016

# Supporting Information

**Materials.**

All reactants and organic solvents were purchased from commercial suppliers. All operations were performed under an argon atmosphere.

**Synthesis of monoadduct and dimer.**

To prepare the monoadduct and dimer of fullerene, we adopt a large excess of C60 with dimethoxy methane to gain a model reaction. Here C60 (200 mg, 0.28 mmol) and dimethoxy methane with molar ratios of 10:1 to give new fullerene derivatives. The mixture was added into anhydrous nitrobenzene (120 mL) and aluminum chloride (448 mg, 3.36 mmol), at 150 °C for 12 hours. Cooling to indoor temperature, the solids were collected by the centrifuge technique, washed repeatedly with cool water, and dried in vacuo at 120 °C. The dried solids were dissolved in a minimum amount of $CH_2Cl_2$ (about 5 mL), and a huge amount of insoluble particles was removed by centrifuge. The clear $CH_2Cl_2$ solution was transferred dropwise into a mixture of 1:1 diethyl ether and hexane (v/v) with vigorous stirring to cause a precipitation of monoadduct. And the solid after centrifugation was a mixture composed of dimer and fullerene molecule.

**Synthesis of the PAF-60-a, PAF-60-b, PAF-60-c, PAF-60, and PAF-60-e.**

C60 (200 mg, 0.28 mmol) and dimethoxy methane with different mole ratios from 1:1, 1:4, 1:8, 1:12, and 1:16 to give PAF-60-a, PAF-60-b, PAF-60-c, PAF-60, and PAF-60-e. The mixture was added into anhydrous nitrobenzene (120 mL), $H_2O$ (2 mL) and aluminum chloride (448 mg, 3.36 mmol), at 150 °C for 72 hours. Cooling to indoor temperature, the resultants were filtered and washed with $H_2O$ (90 mL), $CHCl_3$ (90 mL), acetone (90 mL), and toluene (90 mL) for five times each, followed by drying under vacuum at room temperature. PAF-60-a, PAF-60-b, PAF-60-c, PAF-60, and PAF-60-e were obtained as black powder (about 80 % yields).

**Synthesis of the PAF-61, and PAF-62.**

C60 (200 mg, 0.28 mmol) with acetaldehyde dimethyl acetal and 2, 2-dimethoxypropane (3.36 mmol) was mixed together to give PAF-61, and PAF-62. After repeating the above procedures, PAF-61, and PAF-62 were then obtained in the form of black powders (about 80 % yields).

**Measurements.**

TGA was detected by a Netzch Sta 449c thermal analyzer system in air atmosphere. The FT-IR spectra (KBr, Aldrich) were measured using a Nicolet Impact 410 Fourier transform infrared spectrometer. Samples were packed firmly to get transparent films. The $^{13}C$ MAS NMR experiments were performed on a Bruker AVANCE III 400 WB spectrometer operating at a magnetic field strength of 9.4 T. A small amount of the sample was dispersed in ethanol. After treated by ultrasonic, one drop of the sample mixture was taken from the ethanol solution and transferred to the silicon slice. SEM was performed on a JEOS JSM 6700. A small amount of the sample was dispersed in ethanol. After treated by ultrasonic, one drop of the sample mixture was taken from the ethanol solution and transferred to a 40 copper grid covered by a holey carbon film. TEM was implemented on a JEOL JEM 3010 with an acceleration voltage of 300 kV. Elemental analysis was carried out on a vario Micro and Optima 3300DV elemental analyzer. $N_2$ adsorption isotherms were analysed on a Micromeritics ASAP 2010M analyzer. XRD patterns were obtained on a Riguku D/MAX2550 diffractometer using CuKα radiation. The models of PAFs are constructed by the use of the Materials Studio (MS) simulation environment employing MS Visualizer. The thermogravimetric analysis (TGA) was performed using a Netzch Sta 449c thermal analyzer at the heating rate of 10 $^{o}C$ $min^{-1}$ in dry air atmosphere. It was used to suggest that the decomposition of the skeletons, and there final remains for each PAF. As indicated, the decomposition of the skeletons of all PAFs occurred over 200 °C, and there remains hardly any residue at 600 °C.

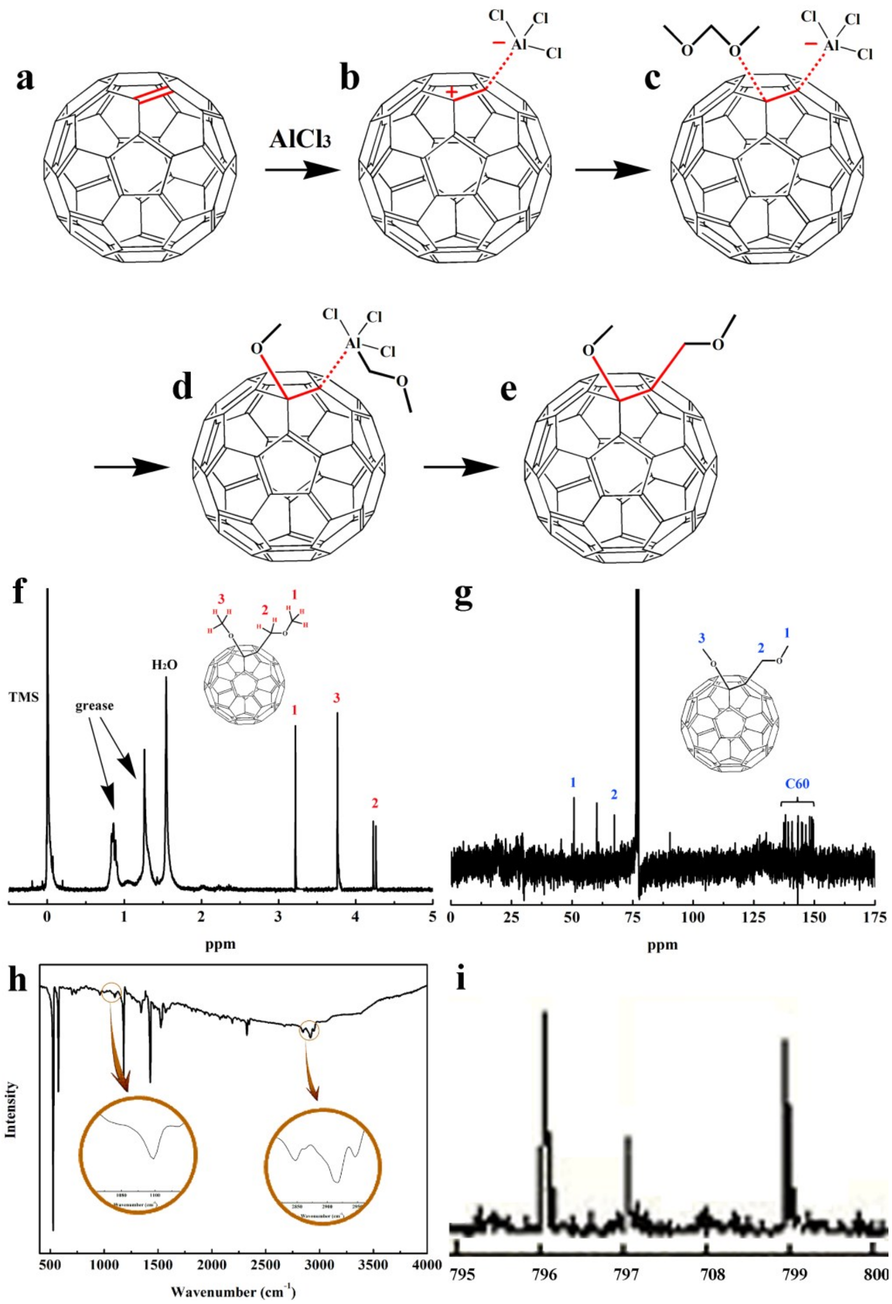


**Fig. S1** Double bond of fullerene molecule could be broken under relatively strong acid catalysis ($AlCl_3$) and fullerene evolved to fullerene cation followed by electrophilic fullerenation of the aromatic. (a-e) the possible reaction mechanism for the acid-catalyzed fullerene to monoadduct; (f) $^1H$ NMR of monoadduct (500 MHz, $CDCl_3$, ppm) δ 4.24 (d, J = 6.8 Hz, 2H), 3.76 (s, 3H), 3.21 (s, 3H); (g) $^{13}C$ NMR spectrum of monoadduct; (h) FT-IR of monoadduct (C-H bands of $-CH_2-$ at 2863 and 2946 $cm^{-1}$ and $-CH_3$ at 2846 and 2916 $cm^{-1}$; C-O band of -C-O- at 1099 $cm^{-1}$); (i) HPLC-ESI/MS image of monoadduct in $CH_2Cl_2$ (Molecule weight of monoadduct is 796).

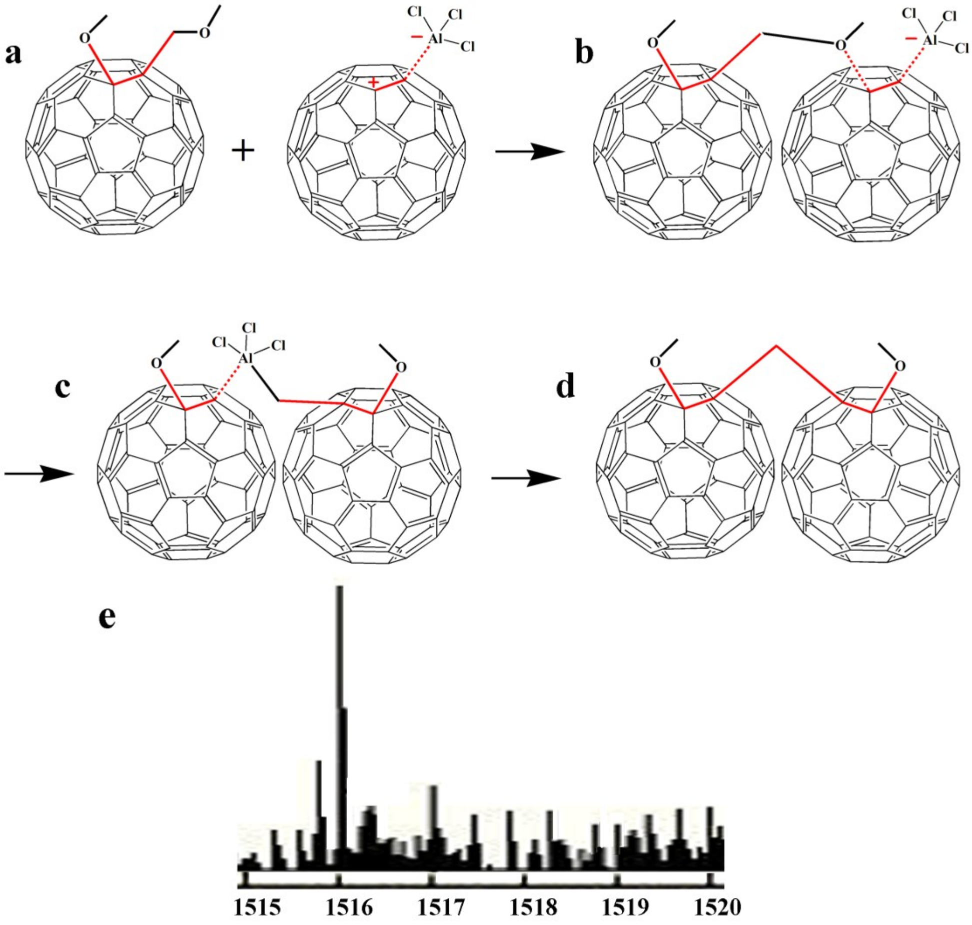


**Fig. S2** (a-d) the possible reaction mechanism for the acid-catalyzed fullerene to dimer; (e) HPLC-ESI/MS image of dimer in $CH_2Cl_2$ (Molecule weight of dimer is 1516).

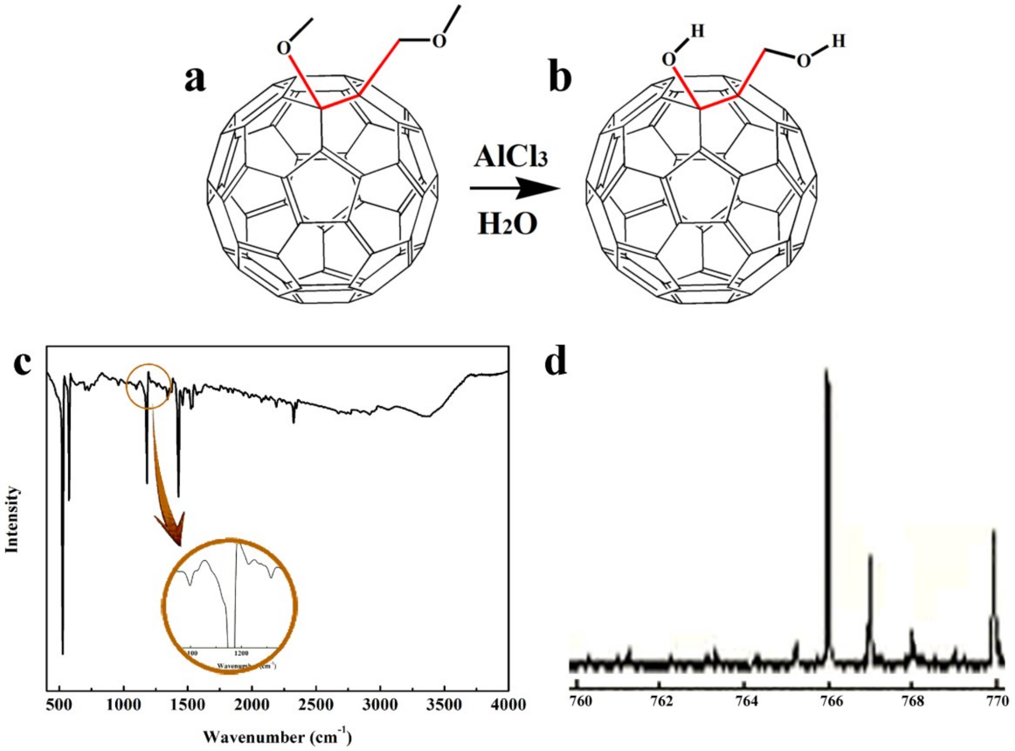


**Fig. S3** The methoxyl group generating into hydroxyl part while the existence of $H_2O$ and $AlCl_3$. (a-b) methoxyl fullerene derivative (monoadduct) become into hydroxyl fullerene derivative. (c) FT-IR of hydroxyl fullerene derivative (C-O bands of –C-OH at 1097 and 1256 $cm^{-1}$; O-H band at 3430 $cm^{-1}$); (d) HPLC-ESI/MS image of hydroxyl fullerene derivative in $CH_2Cl_2$ (Molecule weight of dimer is 768).

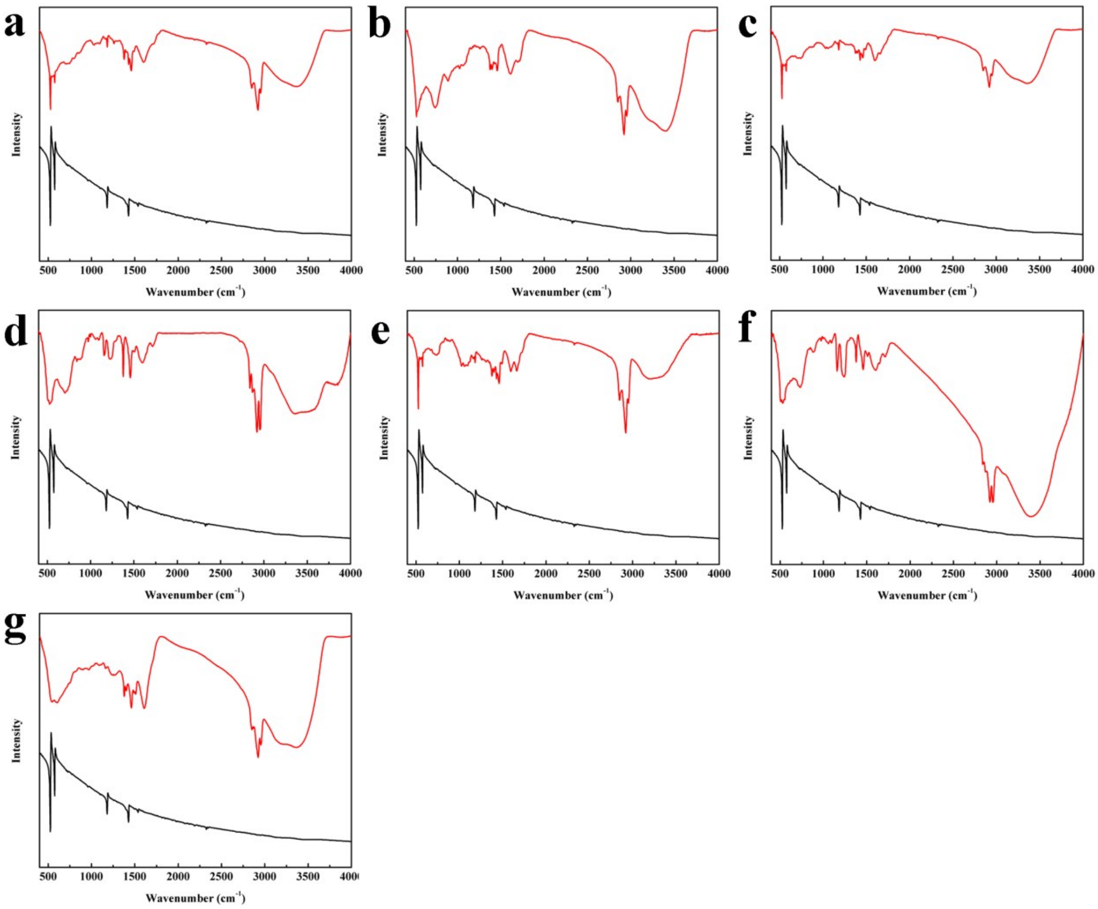


**Fig. S4** FT-IR spectras of PAF-60-a (a), PAF-60-b (b), PAF-60-c (c), PAF-60-e (d), PAF-60 (e), PAF-61 (f), and PAF-62 (g). The C-H vibrations (stretching and bending corresponding to 2950 and 1500 cm$^{-1}$ respectively) of the polymeric frameworks become increasingly obvious with more dimethoxy methane existed in the reaction system. The relative intensity of C=C (1425 cm$^{-1}$) reduced gradually proving the degradation of the conjugation of the original C60.

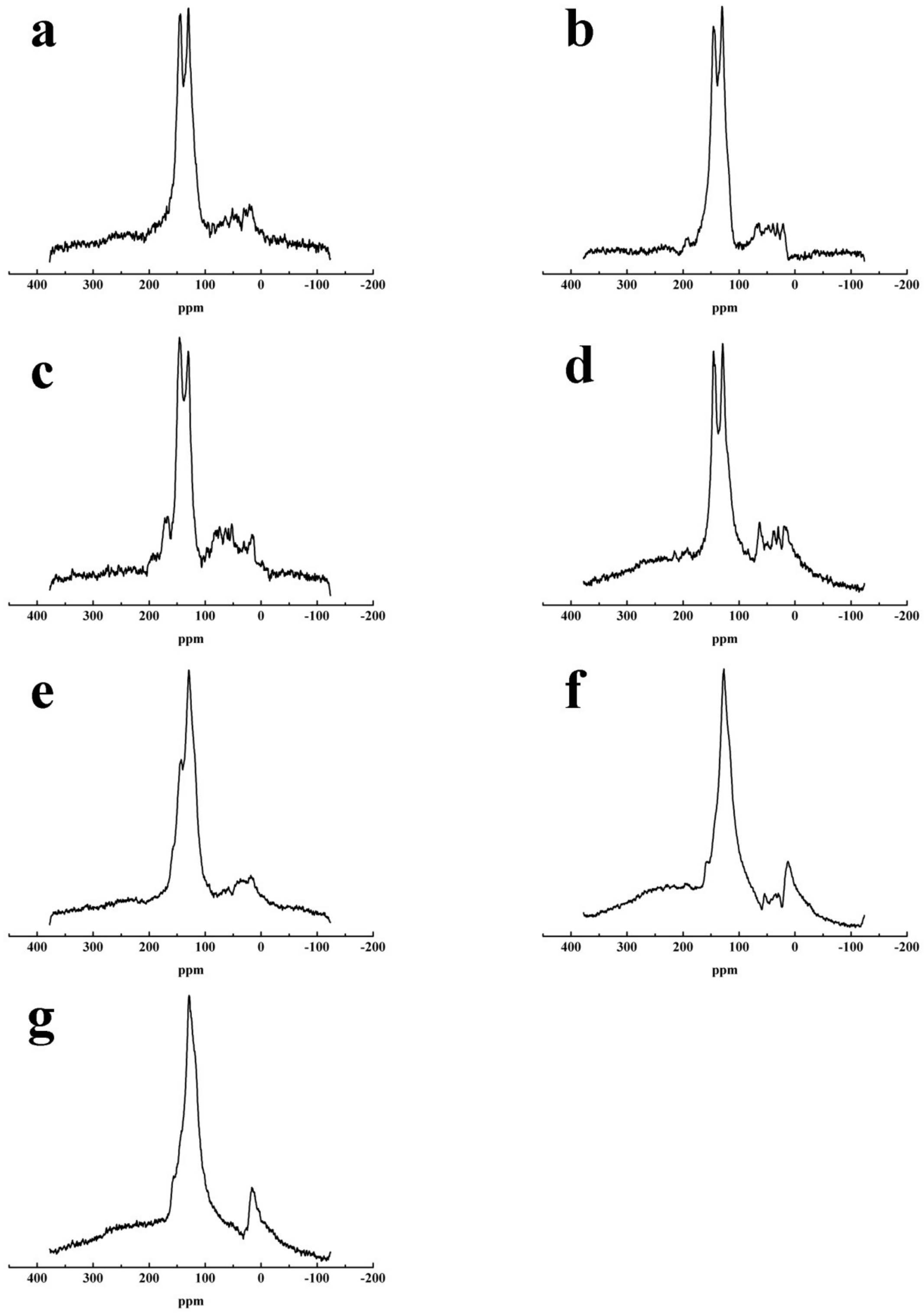


**Fig. S5** Solid-state $^{13}$C CP/MAS NMR spectra of networks PAF-60-a (a), PAF-60-b (b), PAF-60-c (c), PAF-60-e (d), PAF-60 (e), PAF-61 (f), and PAF-62 (g).

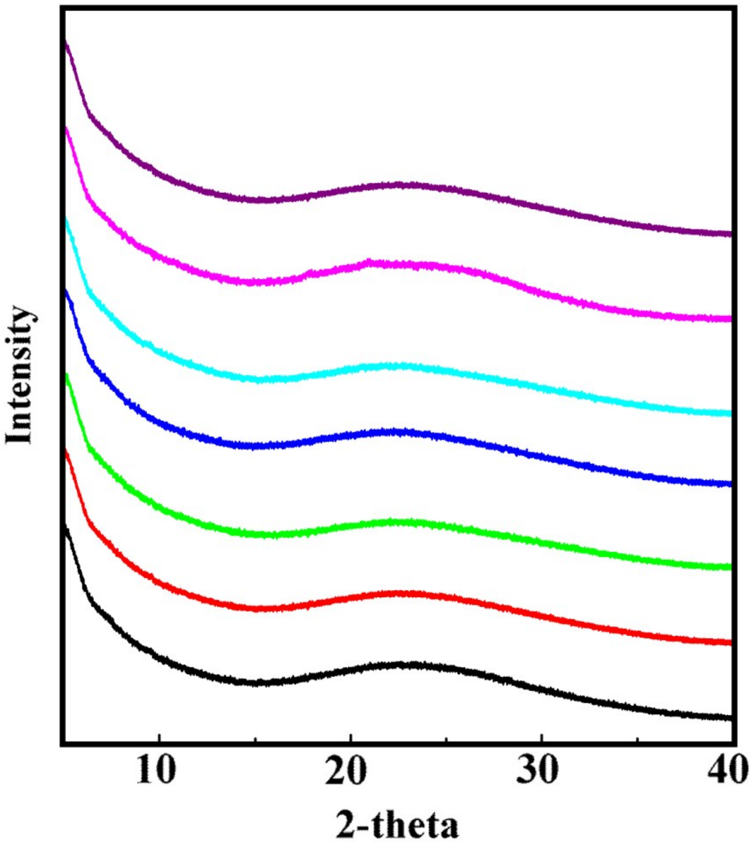


**Fig. S6** XRD patterns of PAF-60-a, PAF-60-b, PAF-60-c, PAF-60-e, PAF-60, PAF-61, and PAF-62. (From down to up)

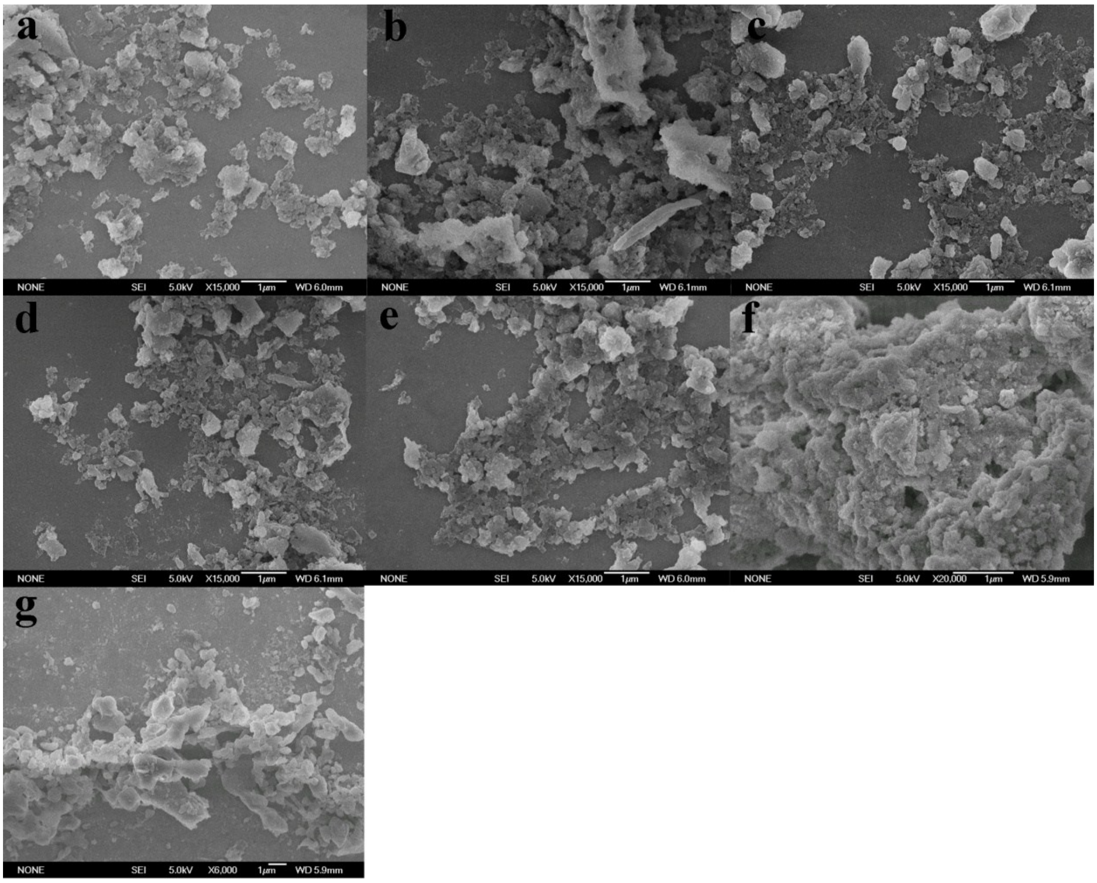


**Fig. S7** SEM images of PAF-60-a (a), PAF-60-b (b), PAF-60-c (c), PAF-60-e (d), PAF-60 (e), PAF-61 (f), and PAF-62 (g).

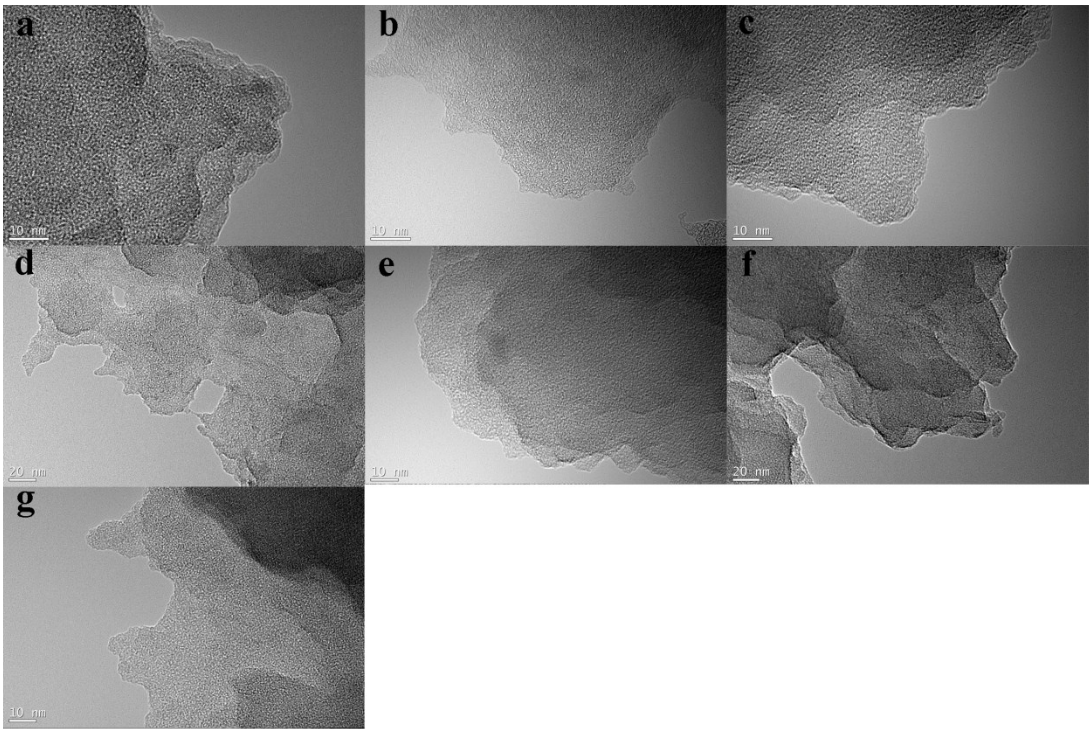


**Fig. S8** TEM images of PAF-60-a (a), PAF-60-b (b), PAF-60-c (c), PAF-60-e (d), PAF-60 (e), PAF-61 (f), and PAF-62 (g).

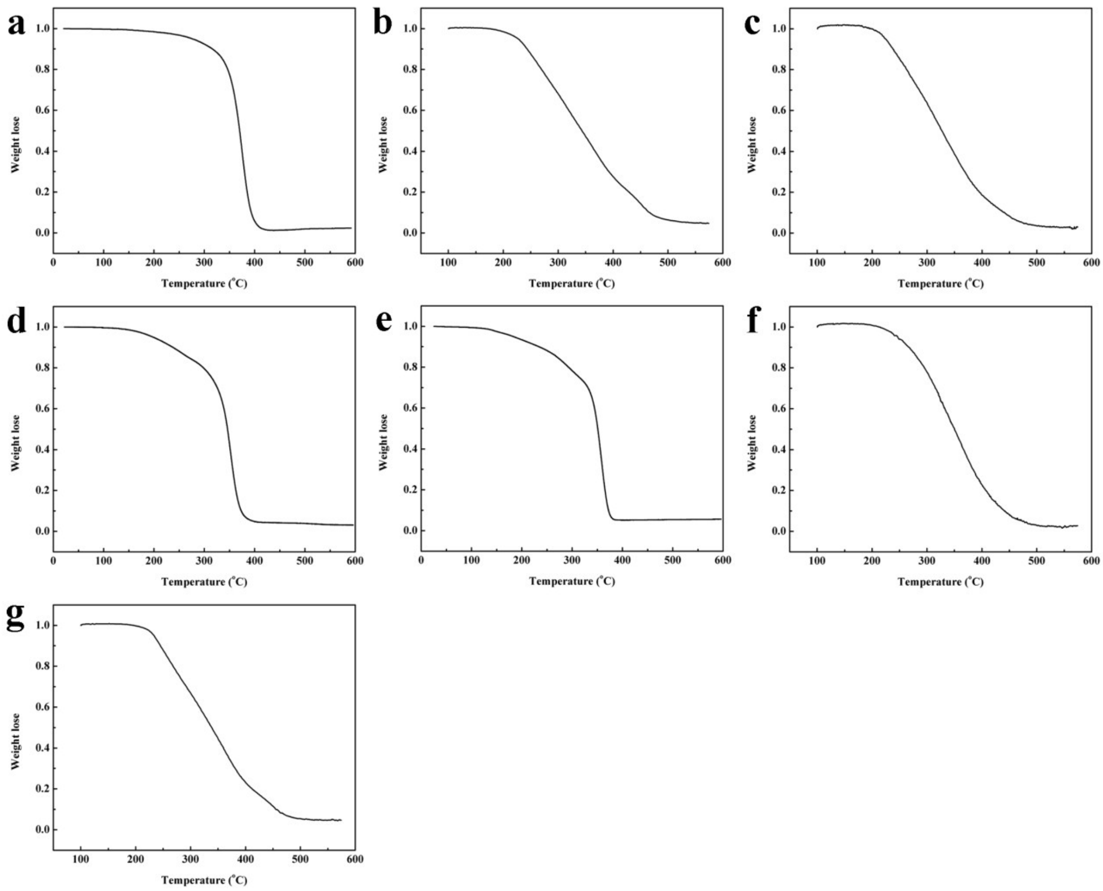


**Fig. S9** TGA curves of PAF-60-a (a), PAF-60-b (b), PAF-60-c (c), PAF-60-e (d), PAF-60 (e), PAF-61 (f), and PAF-62 (g).

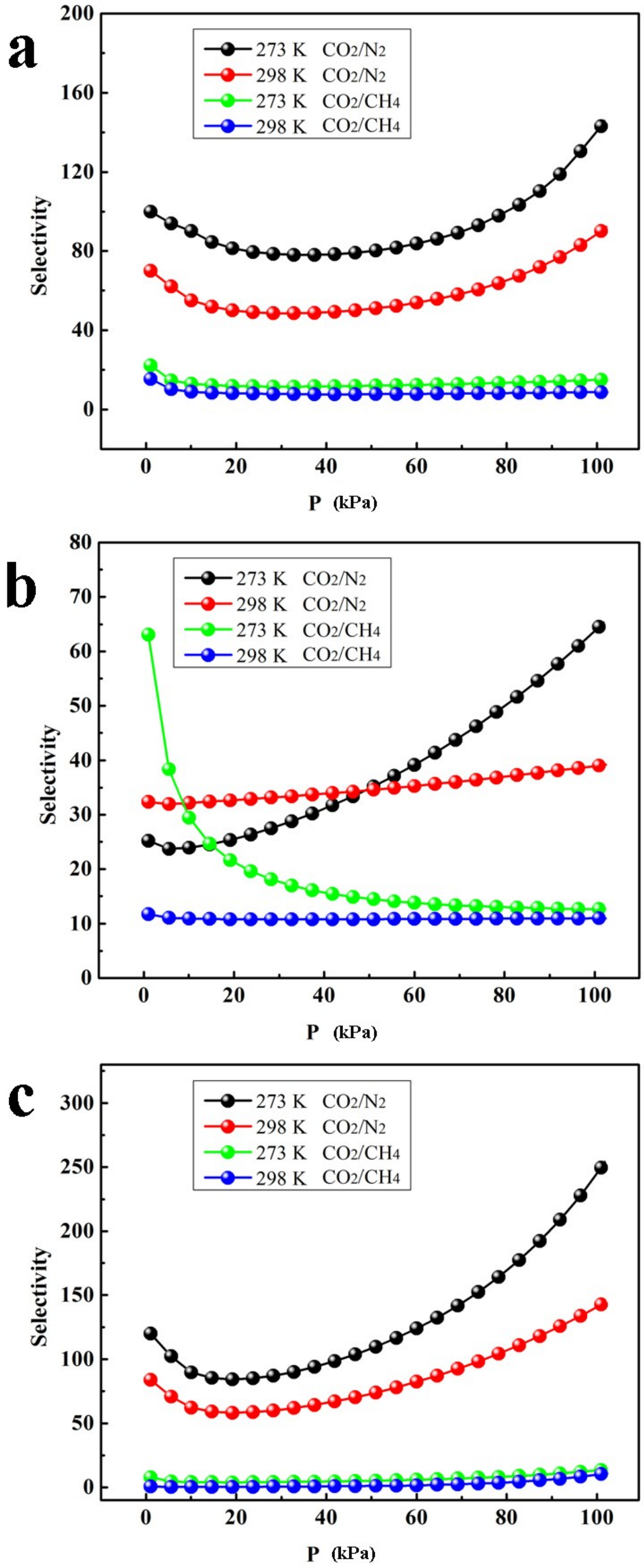


**Fig. S10** Gas selectivity curves of PAF-60 (a), PAF-61 (b), and PAF-62 (c) in IAST method.

**Table S1**

C, H, N element analysis, ICP analysis, and isosteric heats of each PAF.

| | C content (%) | H content (%) | $Al^{3+}$ content (%) | Surface area ($m^2\ g^{-1}$) | $Q_{st}$ $H_2$ (kJ $mol^{-1}$) | $Q_{st}$ $CH_4$ (kJ $mol^{-1}$) | $Q_{st}$ $CO_2$ (kJ $mol^{-1}$) |
|---|---|---|---|---|---|---|---|
| C60 | 100 | 0 | 0 | 19 | 7.4 | 23.9 | 32.4 |
| PAF-60-a | 95.58 | 0.89 | 1.1 | 94/31 | -- | -- | -- |
| PAF-60-b | 85.97 | 2.79 | 2.7 | 350/112 | -- | -- | -- |
| PAF-60-c | 78.74 | 4.25 | 1.4 | 536/408 | -- | -- | -- |
| PAF-60 | 71.44 | 5.71 | 1.6 | 1094/324 | 7.3 | 24.7 | 36.9 |
| PAF-60-e | 73.19 | 5.36 | 3.3 | 852/512 | -- | -- | -- |
| PAF-61 | 77.89 | 5.82 | 1.9 | 793/296 | 7.7 | 21.9 | 31.6 |
| PAF-62 | 76.93 | 6.29 | 2.9 | 701/208 | 7.6 | 24.1 | 34.2 |

The surface area and pore size calculated by $N_2$ sorption isotherms, interpreted by the BET theory and NLDFT method

**Table S2**

$CO_2$ selectivity.

| Material | BET ($m^2$ $g^{-1}$) | $CO_2/CH_4$ | $CO_2/N_2$ |
|---|---|---|---|
| ZIF-95[9] | 1050 | 4.3±0.4 | 18±1.7 |
| ZIF-100[9] | 595 | 5.9±0.4 | 25±2.4 |
| ZIF-78[10] | 620 | 10.6 | 50.1 |
| ZIF-81[10] | 760 | 5.7 | 23.8 |
| ZIF-79[10] | 810 | 5.4 | 23.2 |
| ZIF-69[11] | 950 | 5.1 | 19.9 |
| ZIF-68[11] | 1090 | 5.0 | 18.7 |
| ZIF-82[10] | 1300 | 9.6 | 35.3 |
| ZIF-70[11] | 1730 | 5.2 | 17.3 |
| BPL carbon | 1150 | 3.9 | 17.8 |
| SOF-1a[12] | 474 | 17 | -- |
| PAF-26-COOK[13] | 430 | 8.6 | 50 |
| PAF-26-COOMg[13] | 572 | 8.4 | 73 |
| MOPs-CBZ[14] | 391 | 13.2 | 100 |
| MOPs-DBT[14] | 493 | 10.7 | 80 |
| [Cu(bcppm)$H_2O$][15] | 155 | -- | 590 (15/85 in volume) |
| SIFSIX-2-Cu[16] | 3140 | 5.3 | 13.7 (10/90 in volume) |
| SIFSIX-2-Cu-i[16] | 735 | 33 | 140 (10/90 in volume) |
| SIFSIX-3-Zn[16] | 250 | 231 | 1818 (10/90 in volume) |
| MgMOF-74[17] | -- | -- | 182 (10/90 in volume) |
| NaX zeolite[18] | -- | -- | 146 (10/90 in volume) |
| mmen-CuBTTri[19] | -- | -- | 329 (10/90 in volume) |
| PPN-6-CH2DETA[20] | -- | -- | 442 (10/90 in volume) |
| UTSA-16[21] | -- | -- | 315 (10/90 in volume) |
| PAF-60 | 1094 | 9.81 | 80.4 |
| PAF-61 | 793 | 10.5 | 64.2 |
| PAF-62 | 701 | 18.7 | 275 |
| Measured at 273 K. | | | |

## REFERENCES

[1] J. Polym. Sci. Pol. Chem., 1994, 32, 2727.
[2] J .Am. Chem. Soc., 1991, 113, 9388.
[3] Angew. Chem. Int. Ed., 2007, 46, 3513.
[4] ChemInform, 2010, 31, 2000.
[5] Synthesis, 1989, 5, 372.
[6] J. Chem. Soc. (C), 1970, 227.
[7] J. Chem. Soc. (C), 1970, 109.
[8] Bull. Chem. Soc. Jpn., 1994, 67, 511.
[9] Nature, 2008, 453, 207.
[10] J. Am. Chem. Soc., 2009, 131, 3875.
[11] Science, 2008, 319, 939.
[12] J. Am. Chem. Soc., 2010, 132, 14457.
[13] Polym. Chem., 2014,5, 144.
[14] ACS Appl. Mater. Interfaces, 2014, 6, 7325.
[15] J. Am. Chem. Soc. 2013, 135, 10441.
[16] Nature, 2013, 495, 80.
[17] Proc. Natl. Acad. Sci. U.S.A. 2009, 106, 20637.
[18] Adsorption, 2007, 13, 341.
[19] Chem. Sci. 2011, 2, 2022.
[20] Angew. Chem., Int. Ed. 2012, 51, 7480.
[21] Nat. Commun., 2012, 3, 954.
